# The effect of cadaveric hands-on training model on surgical skills and confidence for transobturator tape surgery

**DOI:** 10.4274/jtgga.galenos.2018.2018.0043

**Published:** 2019-11-28

**Authors:** İlker Selçuk, İlkan Tatar, Emre Huri

**Affiliations:** 1 Clinic of Gynecologic Oncology, University of Health Sciences, Ankara Zekai Tahir Burak Women’s Health Training and Research Hospital, Ankara, Turkey; 2 Department of Anatomy, Hacettepe University Faculty of Medicine, Ankara, Turkey; 3 Department of Urology, Hacettepe University Faculty of Medicine, Ankara, Turkey

**Keywords:** Cadaveric, pelvis, dissection, education, transobturator

## Abstract

**Objective:**

To demonstrate the role of cadaveric hands-on training model on surgical skills and confidence levels of surgeons during transobturator tape (TOT) surgery.

**Material and Methods:**

A pre-test and post-test evaluation to measure skills during the practice of the steps of TOT surgery was performed on a total of 15 postgraduate urologists and gynecologists during a urogynecologic cadaveric dissection course. The course was shaped with regard to theoretical lessons, full pelvic cadaveric dissection and TOT surgery on cadavers.

**Results:**

Good handling of the TOT needle, identifying the right place for groin incision, adequate size of groin incision, identifying the right place for incision at the anterior vagina, dissection of bladder pillars from the vagina, identifying the right place at the vaginal foramina for TOT needle exit, and good placement of mesh were reviewed. The post-test scores were statistically significant for all parameters and also for self confidence level (p<0.001).

**Conclusion:**

Cadaveric workshops are important landmarks of surgical education to improve surgical skills, and gain experience and confidence.

## Introduction

Many residents and young surgeons attend to cadaveric dissection courses, which are in association with surgical anatomy and procedures, because detailed anatomic knowledge is essential for accurate practice in surgical procedures (1,2). Despite learning how to perform surgical procedures, the management of complications and related close anatomic landmarks are lacking in residency. However, it is not unusual to see cadaveric workshops as an integrated part of residency curricula in some gynaecology and obstetrics centres from the first year in residency (3,4).

Postgraduate courses highly focus on improving anatomic knowledge in surgical procedures, and physicians need a good knowledge of anatomy to manage patients intra-operatively and post-operatively. Cadaveric workshops have a beneficial status on the learning points of surgical procedures without any stress of the operation room (5).

Stress urinary incontinence (SUI) is highly common among pre and postmenopausal women, the prevalence is between 4% and 35% (6), and there are many abdominal and vaginal procedures to treat this problem. In general, abdominal procedures such as Burch’s surgery require opening of the Retzius space, which is rich in vascular venous networks and complications may increase morbidity (7). However, a mid-urethral tension-free sling, transobturator tape (TOT) reduces surgical complications (8) and provides adequate support to the urethra in the event of increased abdominal pressure to prevent SUI.

This study aimed to analyse the effect of a cadaveric dissection course on the surgical competence of participants for TOT procedure with a pre/post-test.

## Material and Methods

In total, 15 postgraduate physicians, urologists and gynaecologists, attended this urogynecologic cadaveric dissection course to gain adequate knowledge in pelvic reconstructive surgery and urogynecology. The faculty consists two urologists, one gynaecologist, and one anatomist. After theoretical lessons, a detailed pelvic dissection was performed with two fresh-frozen female cadavers and afterwards a pre/post-test, which measured 8 parameters. The points scale was from 0 (no success) to 5 (very good) and 3 physicians evaluated all participants. First, participants tried to perform a TOT procedure, then mentors showed the tips and tricks of the TOT procedure with step-by-step anatomic landmarks, the participants repeated the procedure and were evaluated for each described intervention (flowchart). One of the check points after the test the measurement of the confidence level of the participants for TOT procedures.

### Statistical analysis

Statistical analyses were performed using the SPSS software version 21. Visual and analytical methods were used to investigate whether the variables were normally distributed. Median and minimum-maximum values were used to present non-normally distributed variables. The Wilcoxon test was used to compare the change in scores between the pre-test and post-test analysis. A p value of less than 0.05 was considered to show a statistically significant result.

### Surgical technique

A 16-F Foley catheter was placed in the bladder, clamped, and tracked to localise the position of the urethra neck. Then, the clitoris was palpated and the parallel location at the genitofemoral fold was used for the groin incision, 0.5-1 cm in length. A mid-urethral vaginal incision 1-1.5 cm in length was performed and, with sharp and blunt dissections, the pubo-cervico-vaginal fascia was opened cranio-laterally until the superposed obturator foramina. After the bladder was removed from the operation field, a TOT needle was inserted from the groin incision (out-in technique) and two clicks was felt while getting into the lateral field of vagina. The pathway of the needle after the skin is the obturator externus muscle, obturator membrane, obturator internus muscle, endopelvic fascia, and vaginal incision. Afterwards, the needle was directed to the vaginal incision to exit. This intervention was performed bilaterally; the tape was laid in front of the urethra with a distance of a Metzenbaum scissor.

## Results

Parameters that were identified to measure the ability for TOT procedure were: good handling of the TOT needle, identifying the right place for the groin incision, adequate size of groin incision, identifying the right place for incision at the anterior vagina, dissection of the bladder pillars from the vagina, identifying the right place at the vaginal foramina for TOT needle exit, and good placement of mesh. At the end, the participants self-evaluated their confidence level as per their situation before the demonstration by mentors and after the course. All parameters were detected as statistically significant (p<0.05) (Table 1).

## Discussion

TOT procedures are very commonly performed by gynaecologists and urologists to increase the quality of life of women due to SUI. It has quite low rates of complications and discomfort after surgery with a desirable improvement in symptoms (9,10). Despite the low complication rates, mesh erosion, sexual discomfort, urinary infection, post-surgery voiding dysfunction, and bladder injury are some complications that may occur after TOT procedures (11,12). Many surgeons need to gain practice and experience in this surgical procedure to feel confident in the operation room and perform better surgeries with increased patient satisfaction.

This study has an important corner, which is in conjunction with urogynecologic practice and anatomic cadaveric studies. It showed that proper surgical education at the cadaveric workshops improved the surgical skills for all steps of a procedure, with increased confidence levels for the operation room.

Surgical training needs a good knowledge of anatomy and clinical consideration for better surgery. In general, there are no formal post-graduate training centres in surgery. However, surgical anatomy education programs should be based on the need of physicians and course directors should plan the flow-chart of the training module according to deficits in surgical anatomy. Additionally, observation and one-to one dissection with mentors, and performing procedures under their guidance increases the basic and advanced anatomic knowledge of participants with clinical concordance (13).

A literature search revealed many anatomic cadaveric studies in the field of urogynecology. Smajda et al. (14) explored the pertinent anatomy during the blind pass of the needle for translevator posterior intravaginal slingplasty. Hubka et al. (15) assessed the tape position in the foramen obturatorum during transvaginal tape (TVT) ABBREVO technique and found no relation of TVT with the obturator nerve. Cadaveric studies were also used in the improvement of anatomic terminology, and the relationship between the external anal sphincter and the bulbocavernous muscle has been investigated previously (16). The functional role of anatomic structures, proximity of anatomic landmarks to the surgical field, and management of probable complications have also been discussed (17,18,19). Moreover, cadaveric dissection studies were compared with anatomic imaging and histologic studies of the human body to improve the detailed knowledge of anatomic structures, so far that will be used in the surgical technique.

This urogynecologic cadaveric dissection course aimed to make surgical procedures easier with cadaveric dissections. Detailed pelvic anatomy with self-practice of participants aimed to increase the topographic view of pelvic and nearby anatomic landmarks during pelvic and urogynecologic surgeries. For surgical approaches, many physicians find it beneficial to attend cadaveric workshops to improve surgical skills in an atmosphere of less stress. This course was planned to be held 3 times per year with 15 participants to improve skills in pelvic reconstructive and urogynecologic surgery.

**Figure 1:**
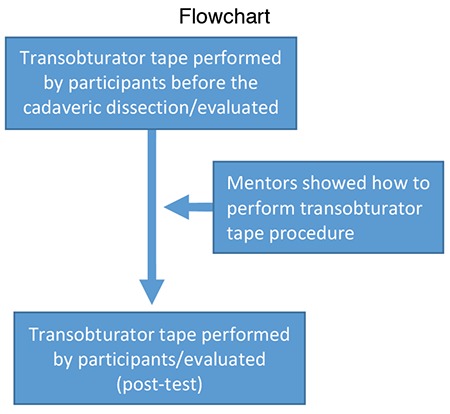
Right pelvic side wall, dissection of obturator space and close anatomic landmarks (star indicates the superposed location where the transobturator tape needle passes)

All the steps of TOT procedure were analysed by mentors and mistakes in the practice of surgery were solved with self-practice. During these steps, close anatomic landmarks were discussed and identified with dissection of the pelvis, perineum, and obturator space. The axis of the trocar needle must be accurate and that needs a good level of experience because the path of the trocar needle is blind and it passes many tissues and fascia around the vessel and nerve structures. Otherwise, the obturator artery, obturator vein or obturator nerve be injured on the lateral pelvic wall, additionally the bladder may also be damaged. Movement of TOT needles were analysed and viewed from the abdominal incision to check probable complications by all participants; the proximity of obturator nerve and obturator foramina, the position of bladder, the location of vagina and endopelvic fascia were evaluated during vaginal dissection and TOT needle insertion (Figure 1). At the end of the course, all participants gained statistically significant surgical skills in performing TOT procedures and had increased surgical confidence levels.

All anatomic steps during TOT surgery (20), place of groin incision, place of vaginal incision, dissection of bladder pillars, TOT needle insertion, path of needle, and placement of mesh were found statistically significant. This infrastructure showed the importance of learning anatomic landmarks for a definitive practice of TOT surgery. Cadaveric courses increase the anatomic knowledge and technical skills, which yields a more confident state for surgeons.

As a conclusion, cadaveric workshops improve basic and advanced anatomic knowledge during the steps of transobturator tape procedure with improved skills and increased confidence status.

## Figures and Tables

**Table 1:**
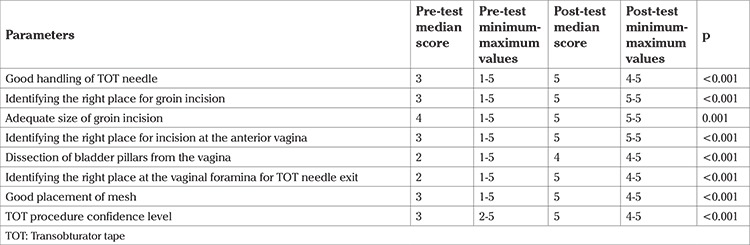
Pre/post-test median scores with minimum and maximum values
